# Supplementary material for: Trends in collisions and traffic mortality rates in Mexico City: A comparison of six data sources
Source: PLoS One. 2025 Oct 7;20(10):e0334103. doi: 10.1371/journal.pone.0334103 (PMC12503325; doi:10.1371/journal.pone.0334103)
Supplement: S1 Appendix — (DOCX) [file pone.0334103.s001.docx]

**S1 Appendix Table 1. Road traffic collisions variables included.**

|  | **ATUS** | **AXA** | **FGJ** | **C5** | **INEGI** | **INCIFO** |
| --- | --- | --- | --- | --- | --- | --- |
| **Type of institution** | **Public, governmental** | **Private** | **Public, governmental** | **Public, governmental** | **Public, governmental** | **Public, governmental** |
| **Road traffic collisions** | **ID_ENTIDAD** 09 | **causa_siniestro** ATROPELLO COLISIÓN Y/O VUELCO **estado** DISTRITO FEDERAL Y/O CIUDAD DE MÉXICO   Duplicate registers were eliminated, filtering by **latitude, longitud, codigo_postal** and **calle** | **ao_hechos** Select the year (2015 – 2022)  **delito** Daños en propiedad ajena culposa por tránsito vehicular a automóvil Daños en propiedad ajena culposa por tránsito vehicular a bienes inmuebles Daños en propiedad ajena culposa por tránsito vehicular a vías de comunicación Lesiones culposas por tránsito vehicular Lesiones culposas por tránsito vehicular por colisión Lesiones culposas por tránsito vehicular por caída de vehículo en movimiento Homicidio culposo por tránsito vehicular Homicidio culposo por tránsito vehicular (atropellamiento), Homicidio culposo por tránsito vehicular (caída) Homicidio culposo por tránsito vehicular (colisión) | **fecha_creacion** Select the year (2015 – 2022)  **codigo_cierre** A | - | - |
| **Non fatal road traffic collisions** | **ID_ENTIDAD** 09  **CLASSACC** Non fatal | **causa_siniestro** ATROPELLO COLISIÓN Y/O VUELCO **estado** DISTRITO FEDERAL Y/O CIUDAD DE MÉXICO   Duplicate registers were eliminated, filtering by **latitude, longitud, codigo_postal** and **calle** | **ao_hechos** Select the year (2015 – 2022)  **delito** Lesiones culposas por tránsito vehicular Lesiones culposas por tránsito vehicular por colisión Lesiones culposas por tránsito vehicular por caída de vehículo en movimiento | **fecha_creacion** Select the year (2015 – 2022)  **tipo_incidente_c4** Accidente Lesionado  **incidente_c4** Atropellado Choque con lesionados Persona atrapada  Persona desbarrancada  **codigo_cierre** A | - | - |
| **Total injuries** | **ID_ENTIDAD** 09 **CLASSACC** No fatal  Addition the total number of: **CONDHERIDO** **PASAHERIDO PEATHERIDO CICLHERIDO OTROHERIDO** | **causa_siniestro** ATROPELLO COLISIÓN Y/O VUELCO **estado** DISTRITO FEDERAL Y/O CIUDAD DE MÉXICO   Duplicate registers were eliminated, filtering by **latitude, longitud, codigo_postal** and **calle** | **anio_hecho** Select the year (2015 – 2022)  **delito** Daños en propiedad ajena culposa por tránsito vehicular a automóvil Daños en propiedad ajena culposa por tránsito vehicular a bienes inmuebles Daños en propiedad ajena culposa por tránsito vehicular a vías de comunicación Lesiones culposas por tránsito vehicular Lesiones culposas por tránsito vehicular por colisión Lesiones culposas por tránsito vehicular por caída de vehículo en movimiento Homicidio culposo por tránsito vehicular Homicidio culposo por tránsito vehicular (atropellamiento), Homicidio culposo por tránsito vehicular (caída) Homicidio culposo por tránsito vehicular (colisión)  **calidad_juridica** LESIONADO  **municipio** CDMX | - | - | - |
| **Fatal road traffic collisions** | **ID_ENTIDAD** 09 **CLASSACC** Fatal | **causa_siniestro** ATROPELLO COLISIÓN Y/O VUELCO **estado** DISTRITO FEDERAL Y/O CIUDAD DE MÉXICO   Duplicate registers were eliminated, filtering by **latitude, longitud, codigo_postal** and **calle** | **ao_hechos** Select the year (2015 – 2022)  **delito** Homicidio culposo por tránsito vehicular Homicidio culposo por tránsito vehicular (atropellamiento), Homicidio culposo por tránsito vehicular (caída) Homicidio culposo por tránsito vehicular (colisión) | **fecha_creacion** Select the year (2015 – 2022)  **tipo_incidente_c4** Accidente Cadáver  **incidente_c4** Accidente automovilístico Atropellado Choque con prensados  **codigo_cierre** A | - | - |
| **Total traffic deaths** | **ID_ENTIDAD** 09  **CLASSACC** Fatal  Addition the total number of: **CONDMUERTO PASAMUERTO PEATMUERTO CICLMUERTO OTROMUERTO** | **causa_siniestro** ATROPELLO COLISIÓN Y/O VUELCO **estado** DISTRITO FEDERAL Y/O CIUDAD DE MÉXICO   Duplicate registers were eliminated, filtering by **latitude, longitud, codigo_postal** and **calle** | **anio_hecho** Select the year (2015 – 2022)  **delito** Daños en propiedad ajena culposa por tránsito vehicular a automóvil Daños en propiedad ajena culposa por tránsito vehicular a bienes inmuebles Daños en propiedad ajena culposa por tránsito vehicular a vías de comunicación Lesiones culposas por tránsito vehicular Lesiones culposas por tránsito vehicular por colisión Lesiones culposas por tránsito vehicular por caída de vehículo en movimiento Homicidio culposo por tránsito vehicular Homicidio culposo por tránsito vehicular (atropellamiento), Homicidio culposo por tránsito vehicular (caída) Homicidio culposo por tránsito vehicular (colisión)  **calidad_juridica** CADAVER  **municipio** CDMX | Not available | **TEMA: DEFUNCIONES**  **CUBO: DEFUNCIONES (INEGI/SS)**  **Causa CIE** V02 – V04.9, V09.2 – V09.9, V12.3 – V14.9, V19.4 – V19.9, V20.3 – V28.9, V29.4 – V29.9, V30.4 – V39.9, V40.4 – V49.9, V50.4 – V69.9, V70.4 – V79.9, V80.3, V80.5, V81.1, V82.1, V83.0 – V88.0, V89.2, V89.9, Y85.0, Y85.9.   **Entidad de Defunción** Ciudad de México  **Fecha Defunción** Select the year (2015 – 2020) | Database provided by an official requisition. Changes did not make. |
