# Supplementary material for: Trends in collisions and traffic mortality rates in Mexico City: A comparison of six data sources
Source: PLoS One. 2025 Oct 7;20(10):e0334103. doi: 10.1371/journal.pone.0334103 (PMC12503325; doi:10.1371/journal.pone.0334103)
Supplement: S2 Appendix — (DOCX) [file pone.0334103.s002.docx]

**S2 Appendix Table 2. Road traffic collision rates in Mexico City from 2015 to 2022.**

| **Data source** | **2015** | **2016** | **2017** | **2018** | **2019** | **2020** | **2021** | **2022** | **2015 – 2020** | **2020 – 2022** |
| --- | --- | --- | --- | --- | --- | --- | --- | --- | --- | --- |
| **Total collisions rate per 1 000 vehicles** | | | | | | | | | | |
| AXA^§^ | 26.51 | 31.06 | 27.70 | 22.39 | 18.74 | 10.18 | 14.00 | 20.74 | -69.3 | 51.1 |
| C5 | 21.94 | 14.84 | 13.88 | 13.08 | 12.41 | 9.24 | 11.54 | 13.72 | -57.9 | 32.6 |
| ATUS | 2.47 | 2.19 | 2.25 | 2.01 | 1.75 | 1.06 | 1.08 | 1.19 | -56.9 | 10.7 |
| FGJ | 1.80 | 1.65 | 1.50 | 1.39 | 1.37 | 1.02 | 1.15 | 1.37 | -43.3 | 25.7 |
| **Collisions resulting in injury rate per 1 000 vehicles** | | | | | | | | | | |
| C5 | 7.87 | 5.03 | 4.68 | 4.57 | 4.33 | 3.46 | 4.42 | 5.67 | -56.0 | 39.0 |
| AXA^§^ | 2.57 | 2.74 | 2.43 | 1.86 | 1.42 | 0.75 | 1.34 | 2.64 | -76.7 | 71.7 |
| FGJ | 0.82 | 0.78 | 0.68 | 0.60 | 0.60 | 0.45 | 0.51 | 0.62 | -45.6 | 27.2 |
| ATUS | 0.43 | 0.42 | 0.34 | 0.36 | 0.36 | 0.20 | 0.23 | 0.26 | -52.5 | 21.2 |
| **Fatal collisions rate per 100 000 collisions** | | | | | | | | | | |
| FGJ | 14.29 | 12.05 | 11.33 | 9.53 | 9.83 | 10.13 | 9.73 | 11.49 | -29.1 | 11.9 |
| C5 | 9.14 | 6.72 | 8.55 | 6.83 | 7.08 | 6.59 | 8.42 | 11.20 | -28.0 | 41.2 |
| ATUS | 4.00 | 4.21 | 3.53 | 3.84 | 3.62 | 2.31 | 2.68 | 3.85 | -42.3 | 40.0 |
| AXA^§^ | 1.02 | 1.03 | 0.40 | 0.70 | 0.55 | 0.39 | 0.30 | 0.35 | -69.2 | -11.0 |
| **Fatality rate per 100 collisions** | | | | | | | | | | |
| FGJ | 7.93 | 7.30 | 7.53 | 6.85 | 7.19 | 9.92 | 8.50 | 8.37 | 25.1 | -18.5 |
| ATUS | 1.62 | 1.92 | 1.57 | 1.91 | 2.06 | 2.17 | 2.48 | 3.23 | 33.7 | 32.8 |
| C5 | 0.42 | 0.45 | 0.62 | 0.52 | 0.57 | 0.71 | 0.73 | 0.82 | 71.0 | 12.7 |
| AXA^§^ | 0.04 | 0.03 | 0.01 | 0.03 | 0.03 | 0.04 | 0.02 | 0.02 | 0.5 | -127.0 |
| **Mortality rate per 100 000 population** | | | | | | | | | | |
| INCIFO | 11.27 | 9.80 | 10.27 | 10.11 | 10.70 | 10.06 | 10.77 | 11.72 | -10.8 | 14.2 |
| INEGI | 8.43 | 7.28 | 7.07 | 5.55 | 4.07 | 6.51 | 6.89 | 6.71 | -22.8 | 3.0 |
| ATUS | 2.32 | 2.51 | 2.24 | 2.65 | 2.50 | 1.66 | 1.90 | 2.84 | -28.3 | 41.4 |
| AXA^§^ | 0.39 | 0.39 | 0.13 | 0.29 | 0.20 | 0.20 | 0.18 | 0.11 | -48.3 | -79.4 |
|  |  |  |  |  |  |  |  |  |  |  |
| § Missing data for October 2021 was imputed with September 2021 data. | | | | | | | | | |  |
