# Supplementary material for: Trends in collisions and traffic mortality rates in Mexico City: A comparison of six data sources
Source: PLoS One. 2025 Oct 7;20(10):e0334103. doi: 10.1371/journal.pone.0334103 (PMC12503325; doi:10.1371/journal.pone.0334103)
Supplement: Attachment — Submitted filename: Response to Reviewers_Herrera.docx [file pone.0334103.s004.docx]

**Response to reviewers**

**Trends in collisions and traffic mortality rates in Mexico City, using different data sources**

We thank the three reviewers for their time, interest, and comments to our paper. We genuinely believe that our paper has been much improved after this revision. Below we provide a point-by-point response to each of the comments.

**Reviewer #1**: This paper offers a unique perspective on traffic safety in Mexico City, addressing a gap in existing research through a comparative analysis of various data sources.

Specific recommendations are as follows:

1. The paper utilizes multiple publicly available datasets for analysis and presents a comprehensive trend analysis of indicators such as collision rates, casualty rates, and fatality rates. The data processing methods align with scientific standards; however, it is recommended that the authors provide a more detailed description of the statistical methods, particularly regarding the handling of missing data and outliers. The current explanation of how missing data are addressed is brief, and a clearer description of how missing values are imputed and how potential duplicate records and outliers are managed would enhance the transparency of the analysis.

Response: Thank you for your comment. We added a paragraph to better explain how we dealt with the data. We did not have to deal with outliers since we used aggregated data - a sum of all collisions or deaths per year and dataset. Visually inspecting the figures, we created (included in the paper), we cannot identify any outlier in the aggregated data.

*The six data sources consist of a nominal list of collisions or deaths with variable information for each event, as described above. Individual records for collisions, collisions resulting in injury and deaths were aggregated (added) per year and dataset. All data sources had available information for the study period except for AXA that was missing information for October 2021. Only in this case, we imputed the monthly value of collisions, collisions resulting in injury and deaths using data from September 2021.*

We also added a sentence regarding elimination of duplicate records in the C5 dataset. Because the source of these data is emergency calls, records can sometimes be duplicated. We followed a systematic process to evaluate duplicates as follows:

*Duplicate records were dropped in two stages. First, we dropped the records with duplication in all variables, except the serial/identification number variable. Second, records were considered duplicates if they shared the same incident code, geographic coordinates, and the reports were one hour or less apart ^1^*

2. In the results section, the authors highlight significant differences in collision and mortality rates across the datasets. However, some paragraphs are dense with information, and the sentences are long and complex. Simplifying the expression and breaking down the trends for each dataset into separate, more digestible segments would improve readability and clarity.

We have reviewed the results section as per your suggestion. We considered reorganizing by dataset but feel that we would lose the comparisons between datasets which is the main objective of our paper. Currently results are organized with this logic: first we describe differences in absolute number of collisions, then collisions resulting in injury, then fatal collisions and then mortality. In the next section we focus on the trends following the same order and finally we briefly discuss strengths and limitations of the data. To avoid too much detail, we give the range highest vs lowest for each of the indicators.

3. The discussion of the reasons behind the dataset differences covers several aspects (e.g., data collection methods, limitations, etc.). It would benefit from being divided into smaller paragraphs, with each topic addressed separately. This would enhance the organization and flow of the section, making it more accessible to readers.

Thank you for this suggestion. We have made several changes to address this. In table 3, we have categorized strengths and weaknesses according to representativeness of the data, mortality definition and other features. We have also added a column called ‘completeness’ where we have ranked the datasets among each other for the different indicators and classified them according to whether they underestimate or are better indicators.

Further, we have reorganized the first section in the discussion. Paragraphs now focus on: differences in data collection methods and informants, mortality definition and coding (for mortality) (see lines starting in 200).

4. The conclusions could further elaborate on the limitations of the data sources. For instance, while the authors mention the underestimation of deaths in the AXA and ATUS datasets, a deeper analysis of the specific reasons for this underestimation, along with additional background information, would provide a more comprehensive understanding of these discrepancies.

Thank you for this comment. We have provided more information about the reasons for the underestimation in Table 3, under limitations -> representativeness, e.g.

*ATUS* ***Representativeness****: Includes a subset of all collisions, only those where a public prosecutor or the police arrives to investigate*

*AXA* ***Representativeness:*** *This is a subset of all collisions because only events occurring among AXA insured vehicles are recorded*

*FGJ* ***Representativeness:*** *Underestimates the total number of collisions because only collisions prosecuted as crimes are included*

*INEGI [difference with INCIFO and FGJ may be due to]* ***:*** *Variable proportion of ill-defined deaths over time because of inconsistencies in coding.*

Then in the discussion in the second and third paragraphs we expand on the limitation of the data sources.

**Reviewer #2:**

My main concern is the research contribution of the paper is marginal and difficult to be identified. It is unconvincing to me the innovativeness and quality of the paper is sufficiently significant to considered to be published in this journal. Here are main issues:

We acknowledge your concern; however, we believe our paper contributes to the literature because we have identified that these data sources are used interchangeably by policymakers, academics and local authorities leading them and the public to often erroneous or biased conclusions. Our paper is the first to discuss limitations of the different datasets.

1. First of all: How does Mexico City law define "road traffic collisions"? An accident is if at least one moving vehicle is involved – is this the case in Mexico City?

Thank you, we have added the definition provided by the Department of Mobility: “A road traffic collision is defined as an avoidable event produced by vehicular traffic, in which at least one vehicle intervenes, causing material damage, injuries and/or deaths.”^2^

2. How is the term "casualties in an accident" defined in your country? Does it refer to a person who died at the scene of the accident, or does it also include deaths occurring within a certain period after the accident (for example, in some countries, it is 30 days after the accident)? If this is also the case in Mexico City, did you take these deaths into account?

Three of the datasets included (police, emergency calls and insurance data) only report casualties that occur at the scene of the accident. The justice department, forensic services and vital statistics include deaths that occur at the scene but also those that occur on the way to hospital, in hospital and at home. The period for classifying the death is not defined (we searched for this information but could not find specifics).

This is part of the reason why we recommend NOT using police, emergency calls or insurance data to report mortality. They vastly underestimate it. The discussion includes a paragraph about this:

*Differences observed across datasets for mortality are due to the mortality definition used, informant, data collection methodology and misclassification of traffic deaths (poor coding). Datasets that only capture deaths at the scene of the collision (such as AXA, ATUS in Mexico) should be avoided to describe traffic mortality. In this study we found that police recorded about 25% of the deaths classified as traffic deaths in INCIFO, while AXA insurance recorded 1% of the deaths recorded by INCIFO….*

3. AXA data definitely cannot be used to assess and analyze the state of safety, given that this database contains only information about vehicles insured by that insurance company. How many such vehicles are there in relation to the total number of vehicles, and are data collected on all accidents (regardless of the consequences)? Why do you consider these data after all?

Thank you. According to the Mexican Association of Insurance Institutions the percentage of insured vehicles in Mexico City is 51.3%. AXA is one of the larger insurance companies but shares the market with many more. AXA registers and records information from all collisions where at least one of its insured vehicles was involved.

You are right that this dataset is not useful to analyze or diagnose the state of safety in the country, however, we find that it is useful to study trends of collisions over time and to evaluate public policy. We have added the following paragraph to the discussion:

In table 3 we have better developed strengths and limitations for this dataset as follows:

*Strengths: Data collection is independent of public services or the government in office and has not changed over time. Collection and completion of the data is linked to insurance claims and payments, which can lead to better care in the collection of information. Collisions are georeferenced. Publicly available. Injuries are classified by level of damage.*

*Limitations:* ***Representativeness:*** *This is a subset of all collisions because only events occurring among AXA insured vehicles are recorded. Therefore, it is not useful to determine the absolute number of collisions or their rates. Key limitation to study trends is that the correct denominator (AXA insured vehicles) is not available. Collisions are mainly mild, with few injuries and deaths.* ***Mortality definition:*** *Deaths are recorded on site, therefore underestimate the true number of deaths among this subset of collisions.*

4. What is the difference between the third and fourth data sources?

The difference between third source (Justice department FGJ, acronym in Spanish) and Police data processed by the National Institute of Geography and Statistics (ATUS for its acronym in Spanish) (fourth source) it is that FGJ only includes collisions prosecuted as crimes. ATUS relies on police reports collected at the scene of the collision which are then validated and harmonized across states. ATUS is a national level dataset while FGJ is city specific.

We have added “city level data source” or “national level data source” for each dataset in the materials and methods section.

5. How do the data from the third and fifth sources differ? In principle, the source of these data is the same – the Justice Department, so how can they differ from each other?

You are right and you have made us look deeper into these datasets. INCIFO data should include 100% of FGJ deaths because INCIFO uses the information in the prosecution files created by FGJ to classify traffic deaths. However, INCIFO reports about 30% more traffic deaths. We have made some enquiries but do not have a confirmed explanation for this. We hypothesize that the FGJ files may sometimes be incomplete e.g. with an unspecific cause of death, and the autopsy performed at INCIFO confirms a traffic collision as the cause of death. We added the following paragraph in discussion:

*We also observe differences in the number of deaths reported by the justice department and the institute of forensic science. A possible explanation for these differences is that the justice department may use unspecific death codes in files which are later confirmed as traffic deaths in the autopsy performed at INCIFO.*

6. What do the codes related to source 6 represent? And based on what criteria were those particular ones selected?

They are International Classification of Disease, 10th Revision (ICD-10) codes related to road traffic collisions. These codes come from death certificates filed by the Health Sector. The codes selected are a standard set commonly used in road safety studies^3 4^. The table below provides the general description for each group of codes

| ICD-10 codes selected | General description |  |
| --- | --- | --- |
| V02 – V04.9 | Pedestrian injured in transport accident except for railway train, railway vehicle or pedal cycle |  |
|  |  |  |
| V09.2 – V09.9 | Pedestrian injured in other and unspecified transportation accidents |  |
|  |  |  |
|  |  |  |
| V12.3 – V14.9 | Pedal cyclist injured in transport accident |  |
|  |  |  |
|  |  |  |
|  |  |  |
| V19.4 – V19.9 | Pedal cyclist injured in other and unspecified transport accidents |  |
|  |  |  |
|  |  |  |
|  |  |  |
|  |  |  |
| V20.3 – V28.9 | Motorcycle rider injured in transport accident |  |
|  |  |  |
|  |  |  |
|  |  |  |
| V29.4 – V29.9 | Motorcycle rider injured in other and unspecified transport accidents |  |
|  |  |  |
|  |  |  |
|  |  |  |
|  |  |  |
| V30.4 – V38.9 | Occupant of three-wheeled motor vehicle injured in transport accident |  |
|  |  |  |
|  |  |  |
|  |  |  |
|  |  |  |
| V39.4 – V39.9 | Occupant of three-wheeled motor vehicle injured in other and unspecified transport accidents |  |
|  |  |  |
|  |  |  |
|  |  |  |
|  |  |  |
| V40.4 – V48.9 | Car occupant injured in transport accident |  |
|  |  |  |
|  |  |  |
|  |  |  |
|  |  |  |
| V49.4 – V49.9 | Car occupant injured in other and unspecified transport accidents |  |
|  |  |  |
|  |  |  |
|  |  |  |
|  |  |  |
| V50.4 – V58.9 | Occupant of pick-up truck or van injured in transport accident |  |
|  |  |  |
|  |  |  |
|  |  |  |
|  |  |  |
| V59.4 – V59.9 | Occupant of pick-up truck or van injured in other and unspecified transport accidents |  |
|  |  |  |
|  |  |  |
|  |  |  |
|  |  |  |
| V60.4 – V68.9 | Occupant of heavy transport vehicle injured in transport accident |  |
|  |  |  |
|  |  |  |
|  |  |  |
|  |  |  |
| V69.4 – V69.9 | Occupant of heavy transport vehicle injured in other and unspecified transport accidents |  |
|  |  |  |
|  |  |  |
|  |  |  |
|  |  |  |
| V70.4 – V78.9 | Bus occupant injured in transport accident |  |
|  |  |  |
|  |  |  |
|  |  |  |
|  |  |  |
| V79.4 – V79.9 | Bus occupant injured in other and unspecified transport accidents |  |
|  |  |  |
|  |  |  |
|  |  |  |
|  |  |  |
| V80 | Other land transport accidents |  |
|  |  |  |
| V81 | Other land transport accidents |  |
| V82 | Other land transport accidents |  |
| V83 - V088 | Other land transport accidents |  |
|  |  |  |
|  |  |  |
|  |  |  |
|  |  |  |
| V89 | Motor - or nonmotor-vehicle accident, type of vehicle unspecified |  |
|  |  |  |
| Y85 | Sequelae of external causes of morbidity and mortality |  |
|  |  |  |

7. What is the difference between insured and registered vehicles? Can a registered vehicle be uninsured, and conversely, can an insured vehicle be unregistered? And why is the number of insured vehicles even considered?

Thank you for your comment. A registered vehicle can be uninsured but not the other way around.

In Mexico, vehicle registration data includes the number of motor vehicles registered by state and municipal governments, classified by type of vehicle and the service they provide

It is not mandatory for vehicles to be insured. According to AMIS (Mexican Association of Insurance Institutions), in 2018, 51.3% of vehicles circulating in Mexico City were insured. Therefore, we believe that using the number of insured vehicles as denominator when using AXA data is a better proxy than using the total number of registered vehicles.

8. Most important: the paper includes a subtitle "Statistical Analysis," but no actual analysis is presented – which statistical method did you use and for what purpose? The title refers to a "trend," but in the paper, apart from the changes in selected indicators from year to year and their percentage differences, we do not see anything else.

We appreciate your point. This is a descriptive paper where the main objective is to highlight the differences and similarities across datasets and to discuss their strengths and limitations. We are not interested in a detailed trend analysis, there are other papers that already do that (see for example ^3 5^). We also note that trends are not linear, therefore while modelling is possible using quadratic terms or splines, the interpretation of modelling estimates is not straight forward. We believe that for the purpose of our study, it is not necessary to include sophisticated models. The visual inspection of graphs is enough to see the very different results obtained from the datasets.

9. The paper lacks an explanation of the motivation for the study. Which databases have been used in previous analyses of traffic safety in Mexico City? Why is there no literature review of previous research – what was used, in what way, and what are the shortcomings of used methods (databases)? Are there studies that research similar questions with usage of different databases?

Based on the above issues, I have to reject this manuscript.

Thank you for your comment. We have added new references to support the following statement:

*These data sources vary in comprehensiveness, methods, and definitions, yet, they have been used indistinctively to assess road safety and to evaluate the impact of interventions in the city ^1 3 5 6^. This issue has been raised by civil society organizations and by governmental institutions but is missing from the academic literature ^7 8^.*

As you can see below, these evaluation studies conducted in Mexico City over the past 10 years all use different data sources.

1. Peón Carballo C, Vadillo Quesada C. Informe "Visión Cero CDMX". Mexico City: ITDP 2018.

**- uses justice department data for mortality and police reports for collisions.**

2. Cárdenas-Cárdenas LM, Barrientos Gutiérrez T, Quistberg DA, et al. One-year impact of a multicomponent, street-level design intervention in Mexico City on pedestrian crashes: a quasi-experimental study. Journal of epidemiology and community health 2022 doi: 10.1136/jech-2022-219335 [published Online First: 2022/12/20]

**- uses emergency calls data for collisions and deaths**

3. Quintero Valverde C, Perez-Ferrer C, Chías Becerril L, et al. Evaluation of road safety policies and their enforcement in Mexico City, 2015-2019: an interrupted time-series study. Injury prevention : journal of the International Society for Child and Adolescent Injury Prevention 2022 doi: 10.1136/ip-2022-044590 [published Online First: 2022/09/13]

**- uses AXA data for collisions and vital statistics for mortality**

4. Chandran A, Perez-Nunez R, Bachani AM, et al. Early impact of a national multi-faceted road safety intervention program in Mexico: results of a time-series analysis. PloS one 2014;9(1):e87482. doi: 10.1371/journal.pone.0087482 [published Online First: 2014/02/06]

**- uses police reports for collisions and vital statistics for mortality**

**Reviewer #3:**

The manuscript "Trends in collisions and traffic mortality rates in Mexico City, using different data sources" examines the trends in road traffic collisions and mortality rates in Mexico City from 2015 to 2022, utilizing six open-access datasets. The study is significant in improving road safety data quality. However, there are areas that need improvement in the methodology, discussion of results, and conclusions.

Thank you for your comments. We have addressed them as described below.

1. Although the paper mentions using multiple data sources to compare the trends in traffic accidents and mortality rates, it does not provide a detailed background on why these four specific indicators (total collision rate, injury-related collision rate, fatal collision rate, and mortality rate) were chosen, or why these six datasets were selected. It would be helpful if the authors could clarify the motivation and significance of these choices in the introduction.

Thank you. We added the following line in the statistical analysis section:

*These indicators are the most widely used by public and private institutions to report locally and nationally all information related to road safety in the country.*

We also added a line and new references in the introduction with respect to the databases:

*These six datasets are the most widely used to evaluate road safety in the city both by academics, civil society and government^1 3 5 6^.*

2. The paper includes several figures illustrating the trend variations across different datasets. However, the captions are somewhat brief, and the explanations for the data changes are not always clear. For example, the magnitude of changes mentioned in Figures 1 and 2 should be further analyzed to understand the underlying causes of these trends. Additionally, the figure titles and explanations in the main text should be more consistent to avoid confusion for the reader. Furthermore, it is recommended to supplement the analysis with additional statistical methods, such as time-series analysis or regression models, to assess trend changes more comprehensively.

We have added some more details to the captions of the figures. We have also changed the color so that the lines are more easily identifiable and have added data labels for the first and last year and for the lowest value in each trend line. We have ensured that colors are consistent across datasets (e.g. ATUS is orange across all figures). It is beyond the scope of our study to understand the underlying causes of the trends. Some possible explanations include changes in the road traffic regulations that occurred in 2015 and 2019 in Mexico City. Other studies have investigated that.^3^

Regarding your comment about further statistical analysis. This is a descriptive paper where the main objective is to highlight the differences and similarities across datasets and to discuss their strengths and limitations. We are not interested in a detailed trend analysis, there are other papers that already do that (see for example ^3 5^). We also note that trends are not linear, therefore while modelling is possible using quadratic terms or splines, the interpretation of modelling estimates is not straight forward. We believe that for the purpose of our study, it is not necessary to include sophisticated models. The visual inspection of graphs is enough to see the very different results obtained from the datasets.

3. In the discussion section, it is recommended to provide specific policy suggestions to help improve the road safety data system in Mexico City, emphasizing the importance of interdepartmental collaboration.

Thank you for this comment and suggestion. We reviewed the legal framework in Mexico City and an important report published in 2018 by a well-respected civil society organization (ITDP) and added a new paragraph to the discussion that reads as follows:

*In Mexico City, the legal framework for a unified and harmonized data source exists^8 9^. For example, in the City’s Road Safety Program, activities such as creating an information system with standardized criteria for road safety and a unique identifier for road safety events are included.^8^ However, institutional fragmentation has made this very difficult. In 2018, the Institute for Transport and Development Policies (ITDP), published a comprehensive report outlining a series of recommendations for an information system, based on their analysis of the data and interviews with key stakeholders^7^. Many of their recommendations are still relevant seven years later. They include creating a data repository in a dedicated platform where the different institutions that generate information on collisions (ATUS, AXA, C5, FGJ, and others) upload their data, harmonizing data collection across institutions using a standardized, digital instrument and training first responders, police, emergency services at hospitals and other actors in its use. It is also recommended that events have a unique identifier so that it is possible to track the incident from the street to its final outcome. Finally, data assurance is recommended to remove duplicate records and avoid coding errors.^7^ Interdepartmental collaboration becomes paramount to be able to design and implement an information system like this. Novel technologies, such as the blockchain could be explored to facilitate recording and tracking of events.10*

4. In the conclusion, the authors highlight that the current datasets do not provide a comprehensive picture of road safety and emphasize the importance of integrating more comprehensive data. While this viewpoint is valid, the authors should further discuss how to gradually transition to a unified information system and the potential impacts and challenges that such a system may bring.

We identified a useful manual published by the World Health Organization that provides recommendations for the design and implementation of data systems^11^. A very detailed description of the design and implementation of a new system is outside the scope of our paper but we have added a paragraph in the discussion section as follows:

*Intersectoral collaboration and funding are paramount to designing and implementing an information system like this. Relevant sectors include law enforcement, transport, city governments, health, emergency services and insurance companies. The initial implementation phase should include training for users, testing of data collection instruments, procedures and software and gradually rolling out^11^. Quality assurance checks of the data should also be done across all implementation stages. An integrated information system will inevitably be challenging to design and implement. Mexico City could look for successful examples elsewhere and replicate what works^11^.*

**References in this document**

1. Cárdenas-Cárdenas LM, Barrientos Gutiérrez T, Quistberg DA, et al. One-year impact of a multicomponent, street-level design intervention in Mexico City on pedestrian crashes: a quasi-experimental study. *Journal of epidemiology and community health* 2022 doi: 10.1136/jech-2022-219335 [published Online First: 2022/12/20]

2. Secretaria de movilidad. Reglamento de Tránsito de CDMX, 2021 [Available from: <https://www.semovi.cdmx.gob.mx/secretaria/marco-normativo/reglamento-de-transito-cdmx-2019> accessed April 2025.

3. Quintero Valverde C, Perez-Ferrer C, Chías Becerril L, et al. Evaluation of road safety policies and their enforcement in Mexico City, 2015-2019: an interrupted time-series study. *Injury prevention : journal of the International Society for Child and Adolescent Injury Prevention* 2022 doi: 10.1136/ip-2022-044590 [published Online First: 2022/09/13]

4. Quistberg DA, Hessel P, Rodriguez DA, et al. Urban landscape and street-design factors associated with road-traffic mortality in Latin America between 2010 and 2016 (SALURBAL): an ecological study. *The Lancet Planetary health* 2022;6(2):e122-e31. doi: 10.1016/s2542-5196(21)00323-5 [published Online First: 2022/02/13]

5. Chandran A, Perez-Nunez R, Bachani AM, et al. Early impact of a national multi-faceted road safety intervention program in Mexico: results of a time-series analysis. *PloS one* 2014;9(1):e87482. doi: 10.1371/journal.pone.0087482 [published Online First: 2014/02/06]

6. Peón Carballo C, Vadillo Quesada C. Informe "Visión Cero CDMX". Mexico City: ITDP 2018.

7. Peón G, Vadillo Quesada C. Sistema de información y seguimiento de seguridad vial: Propuesta de política pública para la Ciudad de México. México: ITDP 2018.

8. Gobierno de la Ciudad de México. Programa Integral de Seguridad Vial 2021-2024 2021 [Available from: <https://www.semovi.cdmx.gob.mx/storage/app/media/PISVI-2021-2024_.pdf> accessed May 2021.

9. Congreso de la Ciudad de México. Ley de Movilidad de la Ciudad de México 2020 [Available from: <https://www.congresocdmx.gob.mx/media/documentos/6299c5bdd0df4f6da6e540ab8613d2682b7d738b.pdf> accessed May 2025.

10. Ahmadisheykhsarmast S, Aminbakhsh S, Sonmez R, et al. A transformative solution for construction safety: Blockchain-based system for accident information management. *Journal of Industrial Information Integration* 2023;35:100491. doi: <https://doi.org/10.1016/j.jii.2023.100491>

11. Social Determinants of Health Team. Data systems: a road safety manual for decision-makers and practitioners. Geneva: World Health Organization 2010.
