# Supplementary material for: Trends in collisions and traffic mortality rates in Mexico City: A comparison of six data sources
Source: PLoS One. 2025 Oct 7;20(10):e0334103. doi: 10.1371/journal.pone.0334103 (PMC12503325; doi:10.1371/journal.pone.0334103)
Supplement: Attachment — Submitted filename: Response to reviewers_R2.docx [file pone.0334103.s005.docx]

**Response to reviewers**

**Trends in collisions and traffic mortality rates in Mexico City: A comparison of six data sources**

**PONE-D-25-05805R1**

**Comments to the Author**

Journal Requirements:

If the reviewer comments include a recommendation to cite specific previously published works, please review and evaluate these publications to determine whether they are relevant and should be cited. There is no requirement to cite these works unless the editor has indicated otherwise. 

Please review your reference list to ensure that it is complete and correct. If you have cited papers that have been retracted, please include the rationale for doing so in the manuscript text, or remove these references and replace them with relevant current references. Any changes to the reference list should be mentioned in the rebuttal letter that accompanies your revised manuscript. If you need to cite a retracted article, indicate the article’s retracted status in the References list and also include a citation and full reference for the retraction notice.

Response: The reviewer comments did not include a recommendation to cite additional works.

We have reviewed the reference list to ensure that it is complete and correct. No changes to the reference list were made in this revision. To our knowledge we have not cited any papers that have been retracted.

Reviewer #1:

Thank you for the authors' careful response to my previous review comments. The authors have made positive improvements in methodology transparency, discussion structure, and analysis of data source limitations, which have enhanced the overall quality of the paper.

Response: Thank you for your comments.

However, I still have concerns about the presentation of the results section. While the authors explained the logic of organizing by indicator type rather than by dataset, which indeed helps maintain consistency in comparisons, the issues of high information density and complex sentence structure have not been fundamentally addressed. Key findings are still buried in lengthy paragraphs, requiring readers to extract main information from complex sentence structures. I suggest the authors further simplify the presentation, consider highlighting important numerical findings in a more prominent way, or add subheadings to break up dense information.

Response: Thank you for your comment. We have restructured the results section by breaking up paragraphs, adding subheadings and ensuring consistent order in the way we reported the results. Please see results section.

Additionally, while the newly added policy recommendations have practical value, the connection between this section and the preceding research findings could be more coherent. I suggest more clearly explaining how these policy recommendations are derived from the research findings to strengthen the logical flow of the argument.

Response: Thank you. We have edited one of the paragraphs in the discussion to better link the policy implications to the results of our study. It now reads as follows:

*…..Based on our findings, the availability of various imperfect datasets implies a major responsibility for data management and processing from recording until their ethical and responsible use.*

*Beyond individuals’ responsibility for appropriate data use, our findings also have policy implications. In Mexico, road safety data systems need to be strengthened, aligning with the World Health Organization’s (WHO) recommendation that governments and relevant stakeholders prioritize the improvement of road safety data collection systems [31]. This requires developing a multisectoral, secure and adaptable information system capable of generating high-quality data for evidence-based policymaking [1]. Strong data collection systems are essential to measure progress towards the Decade of Action for Road Safety (2021 – 2030) targets .*

*In Mexico City, the legal framework for a unified and harmonized data source exists [17, 32]. For example, in the City’s Road Safety Program, activities such as creating an information system with standardized criteria for road safety and a unique identifier for road safety events are included [17]……*

This study fills an important gap in road safety data quality assessment and has good application prospects. I recommend minor revision before acceptance. I hope the authors can focus on optimizing the presentation of the results section to make key findings more easily identifiable and understandable to readers.
